# Non-specific low back pain prevalence and associated factors among Chinese people aged 60 years and older: a cross-sectional study

**DOI:** 10.3389/fpubh.2026.1789937

**Published:** 2026-04-22

**Authors:** Ningjing Chen, Mengnan Chen, Chen Chen, Jiaying Li, Xu Liu

**Affiliations:** 1 School of Nursing, Putian University, Putian, China; 2 Department of Surgical Anesthesiology, Zhongshan Hospital Xiamen University, Xiamen, China; 3 Department of Nephrology, The Fifth Affiliated Hospital Sun Yat-sen University, Zhuhai, China; 4 Faculty of Medicine, The Nethersole School of Nursing, Chinese University of Hong Kong, Hong Kong, Hong Kong SAR, China; 5 Department of Infectious Diseases, The Fifth Affiliated Hospital Sun Yat-sen University, Zhuhai, China

**Keywords:** associated factors, Chinese older people, labor-related factors, lifestyle, non-specific low back pain, prevalence

## Abstract

**Objective:**

This study aims to investigate the prevalence of non-specific low back pain (NLBP) over the past 12 months and to determine its associated factors among Chinese older people aged 60 years and over.

**Methods:**

A cross-sectional study on NLBP prevalence was conducted at seven communities between July 2024 and January 2025. Data on demographic characteristics, lifestyle, and labor-related factors were also collected. This study included those who had suffered from LBP over the past 12 months. The univariate and multivariate Poisson regression analyses were used to examine the factors for the prevalence of LBP.

**Results:**

Of the 286 participants, 129 (45.1%) were females and 174 (60.8%) were in the 60–74 year age group. Overall, the prevalence of NLBP in Chinese people aged 60 and above was 26.2% (95% CI: 21.1, 31.4). Females (adjusted prevalence ratio [aPR]: 6.09, 95% CI: 3.09, 12.00), participants aged 85 years and over (aPR: 1.86, 95% CI: 1.26, 2.73), participants with a body mass index (BMI) ranged from 25.0 and 29.9 kg/m^2^ (aPR: 2.23, 95% CI: 1.29, 3.85), being current smokers (aPR: 4.39, 95% CI: 1.99, 9.69), being former drinkers (aPR: 1.58, 95% CI: 1.04, 2.38), having daily sitting reaching 8 h or longer (aPR: 1.73, 95% CI: 1.26, 2.39), no weekly exercise (aPR: 2.36, 95% CI: 1.50, 3.73), and daily work involving bending (aPR: 2.66, 95% CI: 1.58, 4.48) were associated with NLBP.

**Conclusion:**

NLBP was common in Chinese people aged 60 years and above. Gender, age, BMI, smoking, alcohol drinking, daily sitting reaching 8 h or longer, weekly exercise, and daily work involving bending were associated with the prevalence of NLBP. This study calls for tailored interventions such as launching public health campaigns, organizing social and sporting events, encouraging ergonomic adaptations, and establishing surveillance systems to reduce the LBP burden among Chinese older people.

## 1 Introduction

Low back pain (LBP) refers to pain in the posterior area, ranging from the lower 12th rib margins to the lower gluteal folds, which lasts for at least 1 day (1). LBP has been identified as a leading cause of functional limitations and decreased quality of life globally, with the burden increasing with age (2). A systematic review including 35 studies has concluded that the prevalence of LBP has ranged from 21 to 75% in older people, suggesting a relatively high prevalence among older people (3). Furthermore, it has been projected that the number of people aged 60 years and above will be 2.1 billion by 2050 globally (4). Therefore, the disability burden can increase substantially.

Two-fifths of the LBP burden can be attributed to the associated factors (5). There is an urgent need to examine the associated factors of LBP in the ageing population so that effective prevention and treatment measures can be taken for vulnerable people. Previous studies have shown that demographic characteristics (6–8), lifestyle (9–11), and labor-related factors (12) are associated with LBP. Higher disease burden is found in females (6) and people with a greater age (7). Individuals with a higher body mass index (BMI) have a greater load on the intervertebral disc, contributing to the occurrence of LBP (8). Moreover, smoking accelerates the degeneration of intervertebral discs, contributing to LBP (9). Other lifestyle factors, such as sedentary behavior and lack of exercise, and labor-related factors, for example, daily work involving frequent bending (12), also make contributions to the occurrence of LBP (10, 11).

Although these studies have investigated the prevalence and associated factors of LBP, most of them are conducted in developed countries (7–12). Given that China has a rapidly ageing population and the number of people aged 60 and over is projected to be 402 million by 2040 (13), LBP among Chinese older people becomes a special concern. Even though few studies have investigated the prevalence of LBP in Chinese people, the majority of them are conducted in the working-age population (14). Additionally, the impact of associated factors such as alcohol drinking needs further analysis, as Brauer et al. (15) have shown alcohol drinking is not an associated factor for LBP, while Karimi et al. (16) have suggested it might decrease the likelihood of LBP.

Therefore, a comprehensive investigation on the associated factors is needed. Non-specific low back pain (NLBP) refers to LBP with no specific reason, which accounts for more than 80% of all LBP cases (17). Therefore, this study aims to investigate the prevalence of NLBP over the past 12 months and to determine its associated factors, including gender, age, BMI, smoking, alcohol drinking, occupational factors, sedentary behavior, and exercise among Chinese people aged 60 years and above.

## 2 Methods

This study was reported according to the Strengthening the Reporting of Observational studies in Epidemiology (STROBE) guideline for cross-sectional studies.

### 2.1 Study participants and procedures

This cross-sectional study was conducted at seven communities between July 2024 and January 2025. Two districts were randomly selected from the four districts in Putian. Four communities were randomly selected from each district. However, the head of the community neighborhood committee in one community was unwilling to participate due to time constraints or concerns about privacy. Therefore, seven communities were included in this study. We recruited participants by convenience sampling in each community. Data on demographic characteristics, lifestyle, labor-related factors, and the prevalence of LBP were collected. All participants provided written informed consent before they participated in our study.

### 2.2 Prevalence of non-specific low back pain

In this study, NLBP is defined as pain in the posterior area, ranging from the lower 12th rib margins to the lower gluteal folds with no specific reason, which lasts for at least 1 day. This definition was consistent with the widely accepted clinical criteria (1). For the prevalence of NLBP, we used a question to ask participants about their NLBP experience: “Have you had the experience of NLBP over the past 12 months?” with options of “yes” or “no.”

### 2.3 Demographic characteristics

In this study, we included the following demographic characteristics: gender (male or female), age (60–74, 75–84 or ≥85 years), BMI (<18.5, 18.5 ~ 24.9 or 25.0 ~ 29.9 kg/m^2^), educational level (primary school or less, secondary school, or tertiary or above), and residence (urban or rural area).

### 2.4 Lifestyle factors

Lifestyle factors included smoking (never, former smoker or current smoker), alcohol drinking (never, former drinker or current drinker), daily sitting reaching 8 h or longer (yes or no), and weekly exercise (yes or no). Weekly exercise refers to undertaking at least 150 min of moderate-intensity exercise per week (18).

### 2.5 Labor-related factors

Labor-related factors included the intensity of daily physical labor (light or heavy), daily work involving bending (yes or no), and occupation before retirement (enterprise or institution manager, medical personnel, farmer or factory worker, driver, or housekeeper).

### 2.6 Ethics approval

This study received ethical approval from the Putian University Ethics Committee (approval number: [2024]004, date: March 6, 2024) and was conducted following the Helsinki Declaration. All participants consented to the study. Any information participants provided was kept confidential and accessible exclusively to the research team. All data were anonymised and deidentified.

### 2.7 Statistical analysis

Data analyses were performed using IBM SPSS statistical software version 26.0 for Windows (IBM Corp., Armonk, NY, United States). Data on patients’ socio-demographic characteristics were presented as frequencies (*n*) and proportions (%). The prevalences in males and females by age group were estimated with their 95% confidence intervals (CI). We used the univariate and multivariable Poisson regression models with robust standard errors to examine the associated factors of NLBP prevalence. Previous studies have shown that demographic characteristics (6–8), lifestyle (9–11), and labor-related factors (12) are associated with LBP. Therefore, variables such as demographic characteristics (6–8), lifestyle (9–11), and labor-related factors (12) were selected. These candidate variables were first examined using the univariable Poisson regression, and those with *p* < 0.20 were considered for inclusion in the multivariable model (19). The univariate analyses yielded crude prevalence ratios (PRs), whereas the multivariable logistic regression produced aPRs. To account for potential confounding, we used the aPRs for result reporting, which provide more unbiased estimates of the association between the factors and LBP prevalence. In the multivariable analysis, the model was adjusted for gender, age (years), BMI (kg/m^2^), educational level, residence, smoking, alcohol drinking, daily sitting, weekly exercise, the intensity of daily physical labor, daily work involving bending and occupation before retirement. Prior to conducting the multivariable regression analysis, we also performed collinearity diagnostics to assess potential multicollinearity among the independent variables. The variance inflation factor values for all independent variables ranged from 1.085 to 1.459, which were below 10, indicating the absence of severe collinearity (20). The odds ratios were estimated with their 95% CI in the univariate and multivariate analyses. *p* < 0.05 was considered statistically significant in the multivariate analysis.

## 3 Results

### 3.1 Participants’ socio-demographic characteristics

Table 1 shows the socio-demographic characteristics of participants. We distributed 310 questionnaires to the participants. Only 296 were completed, and 286 questionnaires were valid. The reason for the invalid questionnaires was missing data. In this study, nearly half (45.1%) of the participants were females, and more than 60.0% of the participants were in the 60–74 age group. More than 15.0% of the participants’ BMI ranged from 25.0 to 29.9. Only 19.9% of the participants received tertiary or above education, and more than 60% of them resided in rural areas. Approximately 70% of the participants never smoked or drank alcohol. Additionally, 85.0% of the participants did not sit for 8 h or longer on a daily basis, and 58.4% of them did weekly exercise. Regarding the intensity of daily physical labor, more than 60% of the participants reported light physical labor. However, nearly 70% of the participants engaged in daily work involving bending. In terms of occupation before retirement, 25.2% of the participants were farmers or factory workers, and 30.8% of them were housekeepers.

**Table 1:** Socio-demographic characteristics of study participants.

Variables	Overall (*n* = 286)		NLBP (*n* = 75)	
	*n*	%	*n*	%
Demographic characteristics				
Gender				
Male	157	54.9	15	20.0
Female	129	45.1	60	80.0
Age (years)				
60–74	174	60.8	28	37.3
75–84	87	30.4	29	38.7
≥85	25	8.7	18	24.0
BMI (kg/m^2^)				
<18.5	30	10.5	6	8.0
18.5 ~ 24.9	209	73.1	39	52.0
25.0 ~ 29.9	47	16.4	30	40.0
Educational level				
Primary school or less	152	53.1	46	61.3
Secondary school	77	26.9	14	18.7
Tertiary or above	57	19.9	15	20.0
Residence				
Urban area	112	39.2	22	29.3
Rural area	174	60.8	53	70.7
Lifestyle factors				
Smoking				
Never	212	74.1	58	77.3
Former smoker	40	14.0	6	8.0
Current smoker	34	11.9	11	14.7
Alcohol drinking				
Never	205	71.7	51	68.0
Former drinker	70	24.5	19	25.3
Current drinker	11	3.8	5	6.7
Daily sitting for 8 h or longer				
Yes	43	15.0	29	38.7
No	243	85.0	46	61.3
Weekly exercise				
Yes	167	58.4	16	21.3
No	119	41.6	59	78.7
Labor-related factors				
The intensity of daily physical labor				
Light	176	61.5	25	33.3
Heavy	110	38.5	50	66.7
Daily work involving bending				
Yes	199	69.6	66	88.0
No	87	30.4	9	12.0
Occupation before retirement				
Enterprise or institution manager	35	12.2	6	8.0
Medical personnel	57	19.9	7	9.3
Farmer or factory worker	72	25.2	22	29.3
Driver	34	11.9	11	14.7
Housekeeper	88	30.8	29	38.7

BMI, body mass index; NLBP, non-specific low back pain.

### 3.2 Prevalence of non-specific low back pain

Table 2 presents the prevalence of NLBP. The overall prevalence of NLBP in Chinese people aged 60 and above was 26.2% (95% CI: 21.1, 31.4), which increased with age. The prevalence was 16.1% (95% CI: 10.6, 21.6) in the 60–74 age group, 33.3% (95% CI: 23.2, 43.4) in the 75–84 age group, and 72.0% (95% CI: 53.1, 90.9) in the ≥85 age group. Females had a higher prevalence than males in each age group. For example, the prevalence in females was 31.0% (95% CI: 20.0, 42.0) in the 60–74 age group, which was higher than that of males in the same age group [5.8% (95%:1.2, 10.4)].

**Table 2:** Prevalence of non-specific low back pain.

Age (years)	Prevalence in males (*n* = 157)		Prevalence in females (*n* = 129)		Overall prevalence (*n* = 286)	
	*n*	% (95% CI)	*n*	% (95% CI)	*n*	% (95% CI)
60–74	6	5.8 (1.2, 10.4)	22	31.0 (20.0, 42.0)	28	16.1 (10.6, 21.6)
75–84	6	12.8 (2.9, 22.7)	23	57.5 (41.5, 73.5)	29	33.3 (23.2, 43.4)
≥85	3	42.9 (−6.6, 92.3)	15	83.3 (64.3, 102.4)	18	72.0 (53.1, 90.9)
Overall	15	9.6 (4.9, 14.2)	60	46.5 (37.8, 55.2)	75	26.2 (21.1, 31.4)

CI, confidence interval.

### 3.3 The associated factors of non-specific low back pain

As shown in Table 3, the following factors were associated with NLBP: female (aPR: 6.09, 95% CI: 3.09, 12.00), participants aged 85 years and over (aPR: 1.86, 95% CI: 1.26, 2.73), participants with a BMI ranged from 25.0 and 29.9 kg/m^2^ (aPR:2.23, 95% CI: 1.29, 3.85), being current smokers (aPR: 4.39, 95% CI: 1.99, 9.69), being former drinkers (aPR: 1.58, 95% CI: 1.04, 2.38), having daily sitting reaching 8 h or longer (aPR: 1.73, 95% CI: 1.26, 2.39), no weekly exercise (aPR: 2.36, 95% CI: 1.50, 3.73), and daily work involving bending (aPR: 2.66, 95% CI: 1.58, 4.48).

**Table 3:** The associated factors for non-specific low back pain in Chinese people aged 60 and over (*n* = 286).

Variables	*n* (%)	Univariate		Multivariate	
		Crude PR (95% CI)	*p*	Adjusted PR (95%CI)	*p*
Demographic characteristics					
Gender					
Male	157 (54.9)	Reference		Reference	
Female	129 (45.1)	4.87 (2.91, 8.15)	<0.001	6.09 (3.09, 12.00)	<0.001
Age (years)					
60–74	174 (60.8)	Reference		Reference	
75–84	87 (30.4)	2.07 (1.32, 3.25)	0.002	1.18 (0.82, 1.71)	0.373
≥85	25 (8.7)	4.47 (2.95, 6.80)	<0.001	1.86 (1.26, 2.73)	0.002
BMI (kg/m^2^)					
<18.5	30 (10.5)	Reference		Reference	
18.5 ~ 24.9	209 (73.1)	0.93 (0.43, 2.01)	0.860	1.04 (0.61, 1.77)	0.897
25.0 ~ 29.9	47 (16.4)	3.19 (1.51, 6.74)	0.002	2.23 (1.29, 3.85)	0.004
Educational level					
Primary school or less	152 (53.1)	Reference		Reference	
Secondary school	77 (26.9)	0.60 (0.35, 1.02)	0.060	0.95 (0.61, 1.46)	0.800
Tertiary or above	57 (19.9)	0.87 (0.53, 1.43)	0.581	1.00 (0.62, 1.61)	0.990
Residence					
Urban areas	112 (39.2)	Reference		Reference	
Rural areas	174 (60.8)	1.55 (1.00, 2.40)	0.049	0.87 (0.60, 1.26)	0.464
Lifestyle factors					
Never	212 (74.1)	Reference		Reference	
Former smoker	40 (14.0)	0.55 (0.25, 1.18)	0.126	1.46 (0.73, 2.93)	0.285
Current smoker	34 (11.9)	1.18 (0.69, 2.02)	0.538	4.39 (1.99, 9.69)	<0.001
Alcohol drinking					
Never	205 (71.7)	Reference		Reference	
Former drinker	70 (24.5)	1.09 (0.70, 1.71)	0.705	1.58 (1.04, 2.38)	0.030
Current drinker	11 (3.8)	1.83 (0.92, 3.64)	0.087	1.33 (0.55, 3.22)	0.528
Daily sitting for 8 h or longer					
No	243 (85.0)	Reference		Reference	
Yes	43 (15.0)	3.56 (2.55, 4.97)	<0.001	1.73 (1.26, 2.39)	0.001
Weekly exercise					
Yes	167 (58.4)	Reference		Reference	
No	119 (41.6)	5.18 (3.14, 8.53)	<0.001	2.36 (1.50, 3.73)	<0.001
Labor-related factors					
The intensity of daily physical labor					
Light	176 (61.5)	Reference		Reference	
Heavy	110 (38.5)	3.20 (2.11, 4.86)	<0.001	1.22 (0.85, 1.75)	0.286
Daily work involving bending					
No	87 (30.4)	Reference		Reference	
Yes	199 (69.6)	3.21 (1.68, 6.14)	<0.001	2.66 (1.58, 4.48)	<0.001
Occupation before retirement					
Enterprise or institution manager	35 (12.2)	Reference		Reference	
Medical personnel	57 (19.9)	0.72 (0.26, 1.96)	0.516	0.72 (0.29, 1.77)	0.474
Farmer or factory worker	72 (25.2)	1.78 (0.80, 4.00)	0.161	1.43 (0.69, 2.94)	0.338
Driver	34 (11.9)	1.89 (0.79, 4.53)	0.155	1.72 (0.84, 3.51)	0.140
Housekeeper	88 (30.8)	1.92 (0.88, 4.22)	0.104	1.34 (0.66, 2.73)	0.418

The model was adjusted for gender, age (years), BMI (kg/m^2^), educational level, residence, smoking, alcohol drinking, daily sitting, weekly exercise, the intensity of daily physical labor, daily work involving bending and occupation before retirement. BMI, body mass index; CI, confidence interval; PR, prevalence ratio.

## 4 Discussion

In this cross-sectional study, we estimated the prevalence of NLBP in 286 participants aged 60 years and over selected from seven communities. This study showed that the prevalence of NLBP was 26.2% in Chinese people aged 60 years and above. In the multivariate analysis, gender, age, BMI, smoking, alcohol drinking, daily sitting reaching 8 h or longer, weekly exercise, and work involving bending were associated with the prevalence of LBP.

The prevalence investigated in this study was 26.2%, which was comparable to that of other studies (21, 22). A systematic review and meta-analysis by Wong et al. (21) suggested that the 12-month prevalence rate of non-specific LBP in older adults was 36.1%, which aligns with our findings. Similarly, Williamson et al. (22) found the prevalence rate of LBP was 34.0% in community-dwelling older adults in England, which also supported our findings (22). These results suggested that at least one in four older patients suffered from LBP, which highlighted the need for taking proper measures.

In this study, females reported a higher prevalence compared to males, which is consistent with the previous study (1). Commonly, males and females differed in pain perception and attitudes toward disease. Males were less likely to seek a cure unless the disease worsened (6). For this reason, fewer cases of LBP were reported by males. This study also showed that older people were associated with a higher likelihood of LBP. With increasing age, degeneration occurred in the musculoskeletal system, such as the loss of muscle mass, intervertebral disc degeneration, and reduced bone density, which led to LBP (7). This study also showed that people with a higher BMI were associated with a higher likelihood of LBP. A possible reason might be that a higher BMI was associated with intervertebral disc herniation and spinal stenosis, which contributed to LBP (8).

This study also revealed that smoking was associated with a higher likelihood of LBP, which was consistent with the previous study (23). Smoking decreased blood supply to the intervertebral discs, which accelerated degeneration and caused LBP (9). In addition, proinflammatory cytokines increased in those people who smoked, which sent pain signals to the brain and amplified pain (24). Our study showed that compared to those who never drank, former drinkers were associated with a higher likelihood of LBP. The underlying mechanisms might be that alcohol consumption contributed to muscle cellular metabolic disorders, abnormal inflammatory responses, and body injuries due to impaired coordination (25). Interestingly, this study also suggested the aPR for former drinkers was higher than that for current drinkers. This might be explained by the differences in BMI between these two groups in this study. The proportion of current drinkers with a higher BMI than 24.9 kg/m^2^ was 12.9%, much higher than that of former drinkers (9.1%). People with a higher BMI were at an increased likelihood of LBP. Therefore, the aPR for LBP in former drinkers tended to be higher. Despite this, the effect of drinking has received mixed support in other studies (15, 16). For example, some researchers estimated that alcohol drinking was not associated with the occurrence of LBP by using the Global Burden of Disease Study database (15). In contrast, a systematic review and meta-analysis suggested that alcohol drinking was associated with a lower odds of LBP (16). A potential mechanism was the alcohol-induced release of endogenous opioid ligands, which attenuated the transmission of pain signals to the central nervous system (26). Taken together, future studies aiming at investigating the association between alcohol drinking and LBP are warranted. In this study, people who sit for 8 h or longer and lack weekly exercise were associated with higher odds of LBP. Prolonged upper-body flexion could be commonly seen in sitting postures, which increased spinal loading and caused muscle stiffness and LBP eventually (10). On the contrary, exercise reduced fatty infiltration of the paraspinal muscles and improved muscle contractility and functional capacity, which decreased the occurrence of LBP (11).

This study revealed that people with daily work involving bending were associated with a higher likelihood of LBP. With the degree of flexion of the upper body increasing, the loads on the lumbar spine and muscle activities of the back increased in the same vein (12), which contributed to tissue damage in the spine and back muscles, and caused LBP eventually (27).

Given the growing ageing population, this study highlights the need to reduce the burden of LBP. First, initial efforts from community health service providers are needed to launch public health campaigns and distribute educational materials to raise awareness of these associated factors, including weight management, smoking, alcohol drinking, sedentary behavior, and physical exercise. These initiatives can be implemented through existing community networks such as community health centers and nursing homes to ensure a broad reach (28). In addition, social and sporting events exclusively for the older adults could be organised to promote physical activities. Particular attention should be paid to developing tailored interventions, because such physical activities could be one component of curative and rehabilitative integrated care. Exercise therapy helps relieve pain, promote physical mobility, and improve quality of life, which is one of the most effective strategies for LBP (29). A systematic review and meta-analysis of randomized controlled trials including 989 participants suggested that strength, mind–body and aerobic exercise could greatly relieve LBP in the older adults (29). Concurrently, it is essential to encourage low-cost ergonomic adaptations, e.g., adjustable-height tables in workplaces and households (28), which help to mitigate the impact of ergonomic factors. Furthermore, it can be helpful to establish local or national surveillance systems to monitor the prevalence and determine the cost-effectiveness of different prevention and management strategies (28).

There are several limitations to this study. First, this study is a cross-sectional study, in which we could not estimate the possible causal relationships between the associated factors and the prevalence of NLBP. Therefore, future longitudinal studies are warranted to elucidate the causal pathways. Second, examining the association between the quantified associated factors, e.g., smoking and the prevalence of LBP, provides more insightful findings and strengthens the evidence for the dose–response relationship (30). However, we were unable to perform this analysis in this study due to data limitations. The questionnaire was designed to capture smoking status stratified by smoking habits, that is, never, former smoker, or current smoker at the time of data collection. This study did not gather detailed data on the intensity of smoking, such as cigarettes per day or pack-years, which may be considered in future studies. Third, most of its potential influencing factors were assessed by self-reported data, which were susceptible to recall bias and social desirability bias. Fourth, NLBP was evaluated without clinical evidence. Clinical physical examinations may be conducted for older people in future studies. Fifth, participants in each community were recruited by convenience sampling, which limits the representativeness and generalizability of the study findings. This sampling bias may also affect the prevalence estimates. For example, the number of participants aged >85 years was relatively small, prevalence estimates for this age group might not fully represent the broader population of older adults aged >85 years. Future studies with larger and more representative samples are needed to confirm the findings. Lastly, this study was conducted only in one city in Fujian Province. Therefore, findings in this study may not be applied to other contexts due to the differences in socio-demographics and health systems.

## 5 Conclusion

The prevalence of NLBP was 26.2% in people aged 60 years and above. Gender, age, BMI, smoking, alcohol drinking, daily sitting reaching 8 h or longer, weekly exercise, and daily work involving bending were associated with the prevalence of LBP. To reduce the LBP burden, public health campaigns can be launched to raise older people’s awareness of these associated factors in the communities and nursing homes. In addition, social and sporting events tailored to the needs of older people should be organised to promote physical activities. Low-cost ergonomic adaptations can help to reduce the impact of ergonomic factors. Furthermore, local or national surveillance systems are also needed to determine the cost-effectiveness of different strategies.

## Data Availability

The original contributions presented in the study are included in the article/supplementary material, further inquiries can be directed to the corresponding authors.

## References

[1] Chen N Fong DYT Wong JYH. The global health and economic impact of low-back pain attributable to occupational ergonomic factors in the working-age population by age, sex, geography in 2019. Scand J Work Environ Health. (2023) 49:487–95. doi: 10.5271/sjweh.4116 37634250 PMC10838400

[2] Chen N Fong DYT Wong JYH. Secular trends in musculoskeletal rehabilitation needs in 191 countries and territories from 1990 to 2019. JAMA Netw Open. (2022) 5:e2144198. doi: 10.1001/jamanetworkopen.2021.44198 35044468 PMC8771302

[3] de Souza IMB Sakaguchi TF Yuan SLK Matsutani LA do Espírito-Santo AS Pereira CAB . Prevalence of low back pain in the elderly population: a systematic review. Clinics. (2019) 74:e789. doi: 10.6061/clinics/2019/e789, 31664424 PMC6807687

[4] World Health Organization (2025). Ageing and Health. Available online at: https://www.who.int/news-room/fact-sheets/detail/ageing-and-health (Accessed October 23, 2025)

[5] Ferreira ML de Luca K Haile LM Steinmetz JD Culbreth GT Cross M . Global, regional, and national burden of low back pain, 1990-2020, its attributable risk factors, and projections to 2050: a systematic analysis of the Global Burden of Disease Study 2021. Lancet Rheumatol. (2023) 5:e316–29. doi: 10.1016/s2665-9913(23)00098-x, 37273833 PMC10234592

[6] Carregaro RL Tottoli CR Rodrigues DDS Bosmans JE da Silva EN van Tulder M. Low back pain should be considered a health and research priority in Brazil: lost productivity and healthcare costs between 2012 to 2016. PLoS One. (2020) 15:e0230902. doi: 10.1371/journal.pone.0230902, 32236113 PMC7112211

[7] Blyth FM Noguchi N. Chronic musculoskeletal pain and its impact on older people. Best Pract Res Clin Rheumatol. (2017) 31:160–8. doi: 10.1016/j.berh.2017.10.004 29224694

[8] Segar AH Baroncini A Urban JPG Fairbank J Judge A McCall I. Obesity increases the odds of intervertebral disc herniation and spinal stenosis; an MRI study of 1634 low back pain patients. Eur Spine J. (2024) 33:915–23. doi: 10.1007/s00586-024-08154-4, 38363366

[9] Watkins LR Maier SF. The pain of being sick: implications of immune-to-brain communication for understanding pain. Annu Rev Psychol. (2000) 51:29–57. doi: 10.1146/annurev.psych.51.1.29 10751964

[10] Lee O Park D. Associations between type-specific sedentary behaviour and low back pain: evidence from a large-scale cohort study. Musculoskeletal Care. (2025) 23:e70118. doi: 10.1002/msc.70118 40356065 PMC12069832

[11] Zhang T Firouzabadi A Becker L Schönnagel L Yang D Liu S . Association between lumbar paraspinal muscle activities and quality in chronic low back pain: a cross-sectional analysis. Eur Spine J. (2025) 34:1348–58. doi: 10.1007/s00586-025-08727-x 39988609

[12] Takahashi I Kikuchi S Sato K Sato N. Mechanical load of the lumbar spine during forward bending motion of the trunk-a biomechanical study. Spine (Phila Pa 1976). (2006) 31:18–23. doi: 10.1097/01.brs.0000192636.69129.fb 16395171

[13] World Health Organization. Ageing and Health in China. Available online at: https://www.who.int/china/health-topics/ageing (Accessed October 23, 2025)

[14] Jia N Zhang M Zhang H Ling R Liu Y Li G . Prevalence and risk factors analysis for low back pain among occupational groups in key industries of China. BMC Public Health. (2022) 22:1493. doi: 10.1186/s12889-022-13730-8, 35931976 PMC9354373

[15] Brauer M Roth GA Aravkin AY Zheng P Abate KH Abate YH . Global burden and strength of evidence for 88 risk factors in 204 countries and 811 subnational locations, 1990-2021: a systematic analysis for the Global Burden of Disease Study 2021. Lancet. (2024) 403:2162–203. doi: 10.1016/s0140-6736(24)00933-4, 38762324 PMC11120204

[16] Karimi R Mallah N Nedjat S Beasley MJ Takkouche B. Association between alcohol consumption and chronic pain: a systematic review and meta-analysis. Br J Anaesth. (2022) 129:355–65. doi: 10.1016/j.bja.2022.03.010 35410791

[17] Chiarotto A Koes BW. Nonspecific low back pain. N Engl J Med. (2022) 386:1732–40. doi: 10.1056/NEJMcp2032396, 35507483

[18] Bull FC Al-Ansari SS Biddle S Borodulin K Buman MP Cardon G . World Health Organization 2020 guidelines on physical activity and sedentary behaviour. Br J Sports Med. (2020) 54:1451–62. doi: 10.1136/bjsports-2020-102955, 33239350 PMC7719906

[19] Araoz-Salinas JM Ortiz-Saavedra B Soriano-Moreno AN Reategui-Garcia ME Quispe-Vicuña C Murrieta-Ruiz V . Knowledge and perceptions about diagnosis, clinical management, and prevention of dengue fever among physicians during the 2023 outbreak: a cross-sectional study in Peru. Am J Trop Med Hyg. (2024) 111:1082–92. doi: 10.4269/ajtmh.23-0794, 39191240 PMC11542531

[20] Zhang J Yao C Zheng Y Guo Q. Association between dietary carbohydrate-to-fiber ratio and sleep duration among American adults with constipation: a cross-sectional study. Exp Gerontol. (2026) 216:113066. doi: 10.1016/j.exger.2026.113066, 41679613

[21] Wong CK Mak RY Kwok TS Tsang JS Leung MY Funabashi M . Prevalence, incidence, and factors associated with non-specific chronic low back pain in community-dwelling older adults aged 60 years and older: a systematic review and meta-analysis. J Pain. (2022) 23:509–34. doi: 10.1016/j.jpain.2021.07.012, 34450274

[22] Williamson E Sanchez Santos MT Morris A Garrett A Conway O Boniface G . The prevalence of back and leg pain and the cross-sectional association with adverse health outcomes in community dwelling older adults in England. Spine (Phila Pa 1976). (2021) 46:54–61. doi: 10.1097/brs.0000000000003719, 33315364

[23] Chen N Fong DYT Wong JYH. Health and economic burden of low back pain and rheumatoid arthritis attributable to smoking in 192 countries and territories in 2019. Addiction. (2024) 119:677–85. doi: 10.1111/add.16404, 38105035

[24] Sopori M. Effects of cigarette smoke on the immune system. Nat Rev Immunol. (2002) 2:372–7. doi: 10.1038/nri803 12033743

[25] Zhao Z Lallukka T Chandola T Britton A. Association between alcohol consumption and musculoskeletal pain among employed and retired British civil servants: a multiple group latent class analysis. Eur J Pub Health. (2026) 36:183–92. doi: 10.1093/eurpub/ckaf226, 41406953

[26] Mitchell JM O'Neil JP Janabi M Marks SM Jagust WJ Fields HL. Alcohol consumption induces endogenous opioid release in the human orbitofrontal cortex and nucleus accumbens. Sci Transl Med. (2012) 4:116ra6. doi: 10.1126/scitranslmed.3002902 22238334

[27] Coenen P Kingma I Boot CR Twisk JW Bongers PM van Dieën JH. Cumulative low back load at work as a risk factor of low back pain: a prospective cohort study. J Occup Rehabil. (2013) 23:11–8. doi: 10.1007/s10926-012-9375-z, 22718286 PMC3563950

[28] Khadour FA Khadour YA Alhatem W Albarroush D Halwani AZ Goirge MM . Risk factors of chronic low back pain among Syrian patients: a cross- sectional study. BMC Neurol. (2025) 25:146. doi: 10.1186/s12883-025-04158-9, 40188100 PMC11972510

[29] Zhang SK Gu ML Zhang T Xu H Mao SJ Zhou WS. Effects of exercise therapy on disability, mobility, and quality of life in the elderly with chronic low back pain: a systematic review and meta-analysis of randomized controlled trials. J Orthop Surg Res. (2023) 18:513. doi: 10.1186/s13018-023-03988-y, 37468931 PMC10357808

[30] Schönnagel L Hoehl BU Taheri N Becker L Suwalski P Schömig F . The impact of smoking on low back pain and disability. Eur J Pain. (2025) 29:e70041. doi: 10.1002/ejp.70041, 40392623

